# The Proteoglycan Glypican-1 as a Possible Candidate for Innovative Targeted Therapeutic Strategies for Pancreatic Ductal Adenocarcinoma

**DOI:** 10.3390/ijms231810279

**Published:** 2022-09-07

**Authors:** Davide Busato, Monica Mossenta, Michele Dal Bo, Paolo Macor, Giuseppe Toffoli

**Affiliations:** 1 Experimental and Clinical Pharmacology Unit, Centro di Riferimento Oncologico di Aviano (CRO) IRCCS, 33081 Aviano, Italy; 2 Department of Life Sciences, University of Trieste, 34127 Trieste, Italy

**Keywords:** PDAC, targeted strategies, GPC1, monoclonal antibodies, immunotherapy, nanoparticles, chitosan

## Abstract

Pancreatic ductal adenocarcinoma (PDAC) accounts for 90% of all pancreatic cancers, with a 5-year survival rate of 7% and 80% of patients diagnosed with advanced or metastatic malignancies. Despite recent advances in diagnostic testing, surgical techniques, and systemic therapies, there remain limited options for the effective treatment of PDAC. There is an urgent need to develop targeted therapies that are able to differentiate between cancerous and non-cancerous cells to reduce side effects and better inhibit tumor growth. Antibody-targeted strategies are a potentially effective option for introducing innovative therapies. Antibody-based immunotherapies and antibody-conjugated nanoparticle-based targeted therapies with antibodies targeting specific tumor-associated antigens (TAA) can be proposed. In this context, glypican-1 (GPC1), which is highly expressed in PDAC and not expressed or expressed at very low levels in non-malignant lesions and healthy pancreatic tissues, is a useful TAA that can be achieved by a specific antibody-based immunotherapy and antibody-conjugated nanoparticle-based targeted therapy. In this review, we describe the main clinical features of PDAC. We propose the proteoglycan GPC1 as a useful TAA for PDAC-targeted therapies. We also provide a digression on the main developed approaches of antibody-based immunotherapy and antibody-conjugated nanoparticle-based targeted therapy, which can be used to target GPC1.

## 1. Introduction

**Figure 1:**
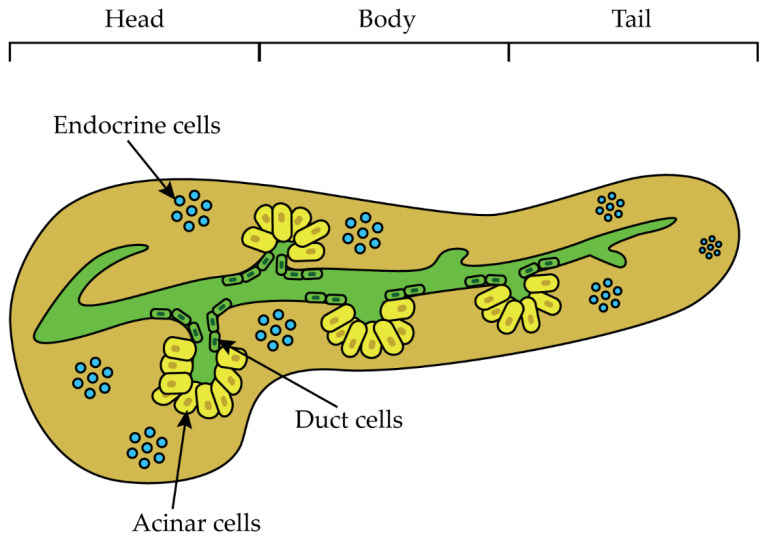
Pancreas anatomy. Macroscopically the pancreas is subdivided into three different parts named the head, body, and tail. Microscopically, it is composed of three main cell types: the endocrine cells designated for the release of hormones, acinar cells, which produce digestive enzymes, and duct cells secreting bicarbonate [4].

Pancreatic cancer is one of the most aggressive and lethal tumor types, with a relative 5-year survival rate of 9% and an increasing number of deaths over the last decade [1]. Pancreatic ductal adenocarcinoma (PDAC) arises in the exocrine region of the organ and accounts for 90% of all pancreatic cancers, with a 5-year survival rate of 7%, the shortest among major cancers [2,3,4]. The NIH estimated a total of 49,830 deaths, 8.2% of all cancer deaths, caused by pancreatic cancer in 2022 in the United States [5]. By 2030, it is likely to be the second leading cause of tumor-related deaths [6,7]. The disease often develops in older adults (>fifty years old), but the incidence is significantly higher in individuals older than seventy years [8]. Most PDAC cases (60–70%) develop from the head of the pancreas and have a slightly better prognosis than lesions arising from the body (15%) and tail (15%) of the organ (Figure 1) [4,9,10].

PDAC is characterized by an initial spread with local diffusion and metastasis to distant organs, with 80% of patients diagnosed in advanced or metastatic stages of the malignancy [11]. Delayed diagnosis is caused by: (i) the absence of specific clinical symptoms, (ii) the impossibility of relying on sensitive and specific markers, (iii) the difficulty of using imaging techniques at early stages, combined with the resistance to conventional therapies, which makes PDAC a malignancy with a high mortality rate. [7,8,12,13]. Surgical intervention is the standard treatment when the treatment has a curative intent. Depending on the possibility of surgical treatment, patients with PDAC can be classified into those with resectable, borderline resectable, non-resectable, and metastatic tumors [14]. On the other hand, chemotherapy and radiotherapy are the two options of systemic treatment for curative or palliative purposes [14]. Despite recent advances in diagnostic investigations, surgical techniques, and systemic therapies, there are still limited options for the effective treatment of PDAC [14]. The currently investigated target therapies for PDAC treatment specifically focus on the various signaling pathways that are altered in the malignancy: RAF-MEK-ERK (MAPK), PI3K-AKT-mTOR, Hedgehog signaling, Wnt signaling, EGFR signaling, and VEGF and VEGFR signaling [4,15]. Nevertheless, there is an urgent need to develop targeted therapies that are able to differentiate between cancerous and non-cancerous cells to reduce side effects and better inhibit tumor growth [4]. Antibody-targeted strategies that can be used as targeted treatments are a potentially effective option for introducing innovative therapies [16,17]. In particular, antibody-based immunotherapy and antibody-conjugated nanoparticle-based targeted therapy can be proposed using antibodies targeting specific tumor-associated antigens (TAA). In this regard, glypican-1 (GPC1), which is highly expressed in PDAC and not expressed or expressed at very low levels in non-malignant lesions and healthy pancreatic tissues, is a useful TAA that can be targeted by specific antibody-based immunotherapy and antibody-conjugated nanoparticle-based targeted therapy [18,19]. In this review, we describe the main clinical features of PDAC. We propose the proteoglycan GPC1 as a useful TAA for targeted therapies of PDAC. We also provide a digression on the main developed approaches of antibody-based immunotherapy and antibody-conjugated nanoparticle-based targeted therapy that can be used to target GPC1.

### 1.1. PDAC Risk Factors

The most important risk factors such as family history, genetic disorders, complications, smoking and alcohol consumption, and eating disorders must be carefully considered to improve the prognosis of patients and to achieve efficient and early detection [7,8,10,20]. It was demonstrated that having a relative with PDAC increases by 6.79-fold the risk ratio of developing the malignancy, and this increases to 9.31 if the relatives are younger than 50 years old [6]. Several inherited syndromes caused by genetic disorders have been described as risk factors for the development of PDAC: familial atypical melanoma syndrome (13÷22-fold increase), hereditary breast and ovarian cancer syndrome (4.1÷5.8-fold increase), Linch syndrome (8.6-fold increase), familial adenomatous polyposis, hereditary pancreatitis (60÷87-fold increase), and Peutz–Jeghers Syndrome (PJS) (132-fold increase) [6,8,20]. The latter syndrome is caused by a germline mutation in the tumor suppressor gene STK11, but the disease manifests only when a second somatic mutation occurs in the STK11 allele [20,21]. Considering the increased risk of developing PDAC in patients with PJS, the National Comprehensive Cancer Network guidelines recommend monitoring these individuals to obtain an early cancer diagnosis [20]. Other risk factors include diabetes mellitus, with a 5.38-fold increase in the risk of developing PDAC within 1 year of diabetes diagnosis, chronic pancreatitis, with a 13.3-fold increase, obesity and smoking, with a 1.68-fold increase, and alcohol consumption, with a 1.22-fold increase [6,8].

### 1.2. Progression

**Figure 2:**
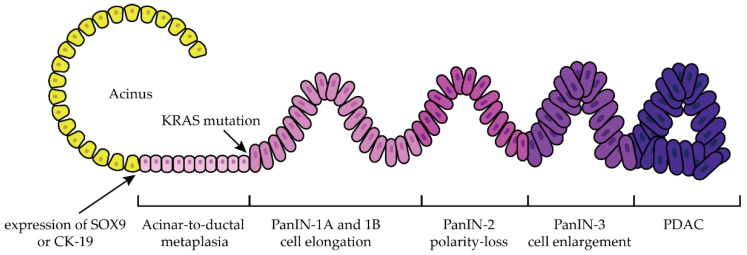
Progression of acinar cells to PDAC. The transition from normal to malignant cells started with ADM. The condition of ADM, as a consequence of KRAS mutation, evolves in pre-cancerous lesions called PanIN. From the initial stages of PanIN-1A and 1B where cells elongate, the malignancy progresses to the PanIN-2 stage characterized by moderate-grade cell lesions and cell polarity loss. Then, cells undergo enlargements, accumulating further genetic mutations until reaching the last stage with the PDAC setting [4].

Evidence suggests that PDAC develops from acinar and/or ductal cells and progresses in a state of chronic inflammation where alternatively activated macrophages recruit regulatory T lymphocytes, creating a highly immunosuppressive microenvironment [22]. Subsequently, cancer-associated fibroblasts produce and release collagen and hyaluronan, increasing interstitial gel fluid pressure and creating an extracellular matrix that impairs blood vessel formation and maintenance, leading to hypoxia [22,23,24]. The following are the three best characterized precursors of PDAC: pancreatic intraepithelial neoplasia (PanIN), intraductal papillary mucinous neoplasms (IPMN). and mucinous cystic neoplasms (MCN) [10,25,26]. Acinar-to-ductal metaplasia (ADM) is considered the parent lesion of PanIN [27]. The generation of ductal-like cells starting from pancreatic acinar cells is the consequence of the ADM process [27]. As a consequence of ADM, the acinar cells showed an enhancement in the expression of typical ductal biomarkers such as sex-determination region Y box 9 (SOX9) or cytokeratin-19 (CK-19), while there is a reduction in the expression in acinar biomarkers such as Mist-1 or amylase [27]. The condition of ADM can be reversible; however, in the presence of particular conditions, such as KRAS mutations, the condition becomes irreversible and progresses to PanIN [27]. PanIN are noninvasive microscopic mucinous papillary lesions less than 5 mm in size that form in smaller pancreatic ducts and are thought to be associated with chronic pancreatitis [10,11]. Continuous cycles of epithelial damage and repair lead to the development of PDAC [10,11]. PanIN lesions are the most common precursors of PDAC and are classified into different stages: PanIN-1A and PanIN-1B, PanIN-2, and PanIN-3, which are characterized by a progressive degree of cellular and nuclear atypia (Figure 2) [4,10,11,26].

IPMN arise from the side branches of the main duct (25%) or from the main duct itself (70%) and are the most common cystic neoplasms detected during surgery [10,11]. They are epithelial tumors that can produce mucin and usually form long finger-like papillae [10,11,25,26]. MCNs account for 25% of resected pancreatic cysts and are characterized by a tumor with mucin-producing epithelial cells and a dense stroma surrounding the cancerous mass [10,11,25]. From a genetic perspective, mutations affecting the proto-oncogene *KRAS* are found in more than 90% of PDAC and are considered the initiating events in PDAC development [8,11,22]. Several cellular signaling pathways and processes are altered in PDAC: KRAS, as previously mentioned, the regulation of G1/S cell cycle transition, homophilic cell adhesion, transforming growth factor-β (TGF-β) signaling, small guanine triphosphate (GTPase)-dependent signaling, the regulation of cell invasion, and integrin signaling [11]. Among these signaling pathways, four genes are frequently mutated and are classified as driver or founder genes: *KRAS* (90%), leading to uncontrolled activation of cell proliferation and survival, *CDKN2A* (90%), which is associated with stimulation of cell proliferation, *TP53* (70%), which overcomes DNA damage and apoptosis checkpoints, and *SMAD4* (55%) which correlates with abnormal TGF-β signaling [11]. Mutations in *CDKN2A*, *TP53*, and *SMAD4* are subsequent of *KRAS* mutations and are a prerequisite for malignancies of invasive pancreatic adenocarcinoma [11]. Moreover, a correlation between the number of mutated founder genes and overall survival as well as disease-free survival has been found [11]. Two models for PDAC evolution were proposed: the first model assumes that PDAC develops from normal epithelium through an accumulation of genetic alterations, starting with the gene *KRAS*, followed by *CDKN2A* and then *TP53* and *SMAD*4 [11]. Starting from pre-tumor cells, clonal expansion occurs with a subsequent increase in mutations, eventually leading to heterogeneity within the tumor, intra-tissular diffusion, and complete tumor development with metastatic diffusion [11]. The second model assumes PDAC evolution based on genomic rearrangements, with most mutations accumulating when the tumor is still diploid or during the preneoplastic state. Then, at least one chromothripsy event occurs, causing copy number changes and evolving into a PDAC [11,28].

### 1.3. Diagnosis, Staging, and Treatment

The symptoms of PDAC are often nonspecific [29]. One study found that of 391 patients suspected of having PDAC, 119 eventually developed the malignancy and had symptoms that were also present in patients who did not have PDAC or who developed other diseases [14,29]. The most common symptoms were loss of appetite, digestive disturbances, and altered bowel habits [14,29]. Some differences can be observed depending on the region of PDAC development; for example, PDAC originating from the pancreatic head causes dark urine, jaundice, loss of appetite and weight, fatigue, and exocrine pancreatic insufficiency [29]. In contrast, PDAC originating from the tail or body of the pancreas presents with abdominal or back pain and symptoms associated with cachexia, which are more nonspecific signs [14,30]. Computed tomography (CT) with angiography of the chest and pelvis is one of the techniques used to diagnose PDAC and assess vascular anatomy and tumor stage [14,31]. The degree of contact between the local blood vessels and the tumor mass determines the initial treatment, so three different stages have been classified: uninvolved, when there is no contact between the tumor and the vessel; adjacent, when the contact covers 180° of the vessel; and enclosed, when the contact is more than 180° [29]. Magnetic resonance imaging and cholangiopancreatography allow for the identification of PDAC metastases in the liver, while positron emission tomography combined with CT and fluorodeoxyglucose as tracers can detect glucose metabolism at the tumor site, allowing for differentiation between benign and malignant lesions, although it is not considered a technique for staging [14]. Endoscopic ultrasonography provides much information for the final cytologic or histologic diagnosis, which is further evaluated by fine-needle core biopsy, an assessment of tumor vascular involvement, an evaluations of lymph node status, and an evaluation of possible complete resection [14]. Serum carbohydrate antigen 19-9 is used as a biomarker for PDAC, especially to monitor tumor responses during treatment, but is not sensitive enough for routine screening [14]. A convenient classification divides PDAC into resectable, borderline resectable, locally advanced, and metastatic to better define available treatment options (Table 1).

A resectable PDAC is characterized by having no or minimal vascular contact with major vessels, and it is treated with resection followed by adjuvant chemotherapy, such as a combination of fluorouracil, oxaliplatin, irinotecan, and leucovinir (FOLFIRINOX) or gemcitabine alone or in combination with capecitabine [14,31]. Borderline-resectable PDAC has venous and/or partial arterial involvement and requires neoadjuvant therapy to increase the number of resectable patients, whereas radiation is recommended for unresectable PDAC without metastases [14]. In locally advanced PDAC, vascular invasion is present, and surgery is not feasible. The therapeutic options available for this stage are an initial chemotherapeutic treatment with modified FOLFIRINOX or albumin-bound paclitaxel and gemcitabine [14,32]. For metastatic disease, the regimen is modified FOLFIRINOX (the dose of irinotecan is reduced from 180 mg/m^2^ to 150 mg/m^2^) or albumin-bound paclitaxel and gemcitabine. For a subset of patients with BRCA1/2 germline variations, a platinum-based first-line treatment with olaparib was approved, extending median progression-free survival from 3.8 months (placebo) to 7.4 months [14,31,33]. Patients previously treated with gemcitabine may receive a combination of 5-fluorouracil plus leucovorin and nanoliposomal irinotecan as a second-line therapy. This strategy prolonged median overall survival from 4.2 months (5-fluorouracil plus leucovorin) to 6.1 months without further safety concerns. Unfortunately, therapeutic treatments have several drawbacks, with grade 3 and 4 adverse effects leading to treatment discontinuation [25,31]. One of the possible causes of this toxicity is that conventional therapies interact with various cellular processes without the ability to distinguish between malignant and healthy cells, resulting in the various and severe adverse effects [4]. Furthermore, the lack of effective treatments is compromised by the presence of mechanisms of drug resistance [34]. To date, almost 165 genes have been found to be related to drug resistance in PDAC [34]. Innumerable cell functions, such as cell cycle regulation, metastasis, antioxidant activity, apoptosis, and signal transduction, have been associated with these genes [34]. The two glycoprotein families of ABC transporters and mucin proteins play a pivotal role in drug resistance [34]. ABC transporters, specific proteins delegated to the secretion of the drug outside the cells, are frequently overexpressed in PDAC [34]. High expression of mucin proteins is associated with the expression of genes involved in drug resistance [34]. miRNA also play an important role in drug resistance by regulating genes involved in cell proliferation, invasion, and metastasis [34]. For example, the overexpression of microRNA 181c is thought to induce the activation of YAP and TAZ, the main effectors of the HIPPO pathway, leading to drug resistance [35].

Lastly, cancer stem cells play a key role in drug resistance through the overexpression of ABC transporter and other enzymes implicated in drug metabolism and DNA damage repair [34]. Considering the resistance to nab-Paclitaxel, metabolic alterations were observed in PDAC cell lines [36]. A study conducted on PDAC cell lines demonstrated that the resistance to nab-Paclitaxel is presumably related to the up-regulation of polyol pathway, a pathway involved in the catabolism of glucose [36]. Based on this information, a therapeutic regimen of nab-Paclitaxel coupled with an aldose-reductase inhibitor could improve the efficacy of nab-Paclitaxel [36]. Another metabolic alteration that may be related to nab-Paclitaxel resistance is represented by the enhancement in lactate concentration [36]. The last evidence of metabolism alteration is related to an enhance in carbamoyl-aspartic acid, a compound necessary for pyrimidine synthesis, and a decrease in aspartic acid [36]. This observation can be justified by an enhancement of the activity of the aspartate transcarbamylase, the enzyme responsible of the formation of carbamoyl-aspartate, starting from aspartic acid [36]. The availability of elevated amounts of pyrimidines could cause the onset of drug resistance mechanisms [36]. Considering this clinical situation, the genetic and molecular profile must be considered for the development of new and efficient targeted therapeutic strategies [37]. Several studies show that the cell surface proteoglycan GPC1 is a potential therapeutic and diagnostic protein for PDAC [38,39,40,41].

## 2. Glypican-1 as Target Protein

### 2.1. Glypican Family

Glypicans and syndecans are the two main components of the proteoglycan family [42]. Proteins belonging to this family consist of a core protein to which several glycosaminoglycans (GAG) can be attached at specific sites [43]. Proteoglycans are found attached at the cell membrane and are also one of the main components of the extracellular matrix (ECM) [43]. Proteoglycans play a role in the developmental process; in particular, they are able to coordinate signaling molecules released in the extracellular space (members of Hedgehog, Wingless (Wnt/Wg), fibroblast growth factor (FGF), bone morphogenetic protein (BMP), and TGF-β), which can trigger the intracellular signal transduction cascades responsible for the shape and structure of pluricellular organisms [43].

Glypicans are bound to the cell membrane via a glycosylphospatidylinositol (GPI) anchor, but can be released from the cell surface by enzymatic cleavage [44,45]. These proteins have a general structure with a size of 60–70 kDa, consisting of an N-terminal secretory signal peptide, a hydrophobic domain essential for the GPI anchor, and heparan sulfate (HS) chains linked to the C-terminal domain near the cell surface [44]. The HS chains originate from a precursor synthesized in the Golgi as a post-translational process [46]. HS chains can have different lengths and can be modified by deacetylation, sulfation, and epimerization [47]. Sulfation confers a negative charge that facilitates interactions between HS and positively charged proteins such as heparin-binding growth factors and BMPs [47].

In mammals, the glypican family consists of six members that can be divided into two subfamilies: glypican 1/2/4/6 and glypican 3/5 [47]. Homology between glypicans is high, especially in the N-terminal regions, and the presence of 14 conserved cysteine residues makes the three-dimensional structure very similar within the glypican family [44,48]. Glypicans are mainly expressed during development and play a key role in developmental morphogenesis [44,49]. The regulation of developmental morphogenesis occurs through interactions, probably by HS chains with morphogens, coordinating their cell surface levels in a concentration-dependent manner [43,47]. A study conducted on adenocarcinoma cells demonstrated that the HS chains of glypicans interact with matrix metalloproteinase-9 (MMP-9), playing a pivotal role in cell motility [50].

In adult tissues, GPC1 is highly expressed in the testis, whereas it is expressed at low levels in the heart, kidney, ovary, placenta, adrenal gland, and thyroid, and is very low or undetectable in the pancreas, liver, lung, stomach, small intestine, prostate, colon, brain, esophagus, and thymus; glypican 2 (GPC2) is absent in all adult tissues; glypican 3 (GPC3) expression is low and restricted to the ovaries, mammary gland, mesothelium, lung, and kidney; glypican 4 (GPC4) is detectable in many tissues; glypican 5 is restricted to the brain, kidney, and liver; glypican 6 (GPC6) is expressed in various tissues [18,49,51,52,53,54,55,56,57].

Interestingly, several studies reported aberrant expression of glypican during tumor progression [44].

GPC1 protein expression levels are high in pancreatic cancer, squamous cell carcinoma of the esophagus, prostate cancer, breast cancer, and gliomas [18,38,58,59,60,61]. GPC2 is highly expressed in several pediatric tumors, including neuroblastoma [51]. Hepatocellular carcinoma (HCC) is the most studied cancer in which GPC3 is highly expressed [62]. Other tumors associated with GPC3 include squamous cell carcinomas of the lung, non-seminomatous germ cell tumors of the testis, liposarcomas, melanomas, ovarian carcinomas, neuroblastomas, Wilms tumors, and rhabdomyosarcomas [63,64,65,66,67]. Pathways such as Wnt, Hedgehog, and YAP are often dysregulated in several types of cancer, and GPC3 has been shown to play a key role in the coordination of these pathways, even if it is not still well defined in several cases [68]. GPC4 is involved in insulin resistance, body fat distribution, and non-alcoholic fatty liver disease [69]. GPC5 is highly expressed in rhabdomyosarcomas and promotes a high proliferation rate in the cells of this cancer, while GPC5 acts as a suppressor of tumor growth in the context of non-small-cell lung cancer cells [70,71,72]. The expression of GPC6 is high in gastric adenocarcinomas compared to healthy tissues, although its role in tumorigenesis needs further investigation [73].

### 2.2. Glypican-1

**Figure 3:**
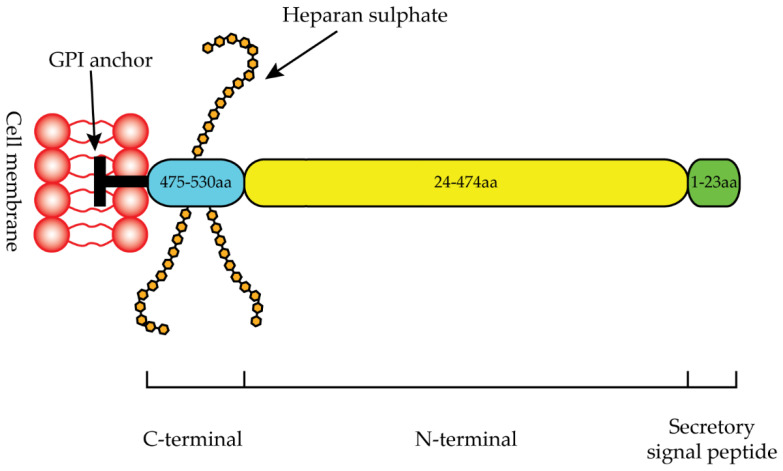
Schematic representation of GPC1 protein.

GPC1 was the first member of the HS proteoglycans to be discovered [74]. The *GPC1* gene is located on chromosome 2 (2q37.3) and consists of 32,381 base pairs of genomic DNA with nine exons [75]. The GPC1 protein consists of 558 amino acids with a molecular weight of 62 kDa [75]. The first 23 amino acids represent the secretory signal peptide, the N-terminal region ranges from 24 to 474 amino acids, and the C-terminal region ranges from 475 to 530 (Figure 3) [76]. The C-terminal domain terminates with a hydrophobic region essential for GPI anchor linkage (Figure 3) [76].

Two N-linked glycans are linked to Asn-79 and Asn-116, and three HS chains are also linked to Ser-486, Ser-488, and Ser-490 [76]. Six disulfide bonds are present in the N-terminal region, and two additional cysteine residues are located in the C-terminal region, where they may be free or present as disulfide bonds [77].

As mentioned earlier, GPC1 expression is very limited in most adult tissues [18,78]. In the embryonic context, GPC1 is normally expressed [78]. In vivo studies have shown that GPC1 is required for physiological brain development. In addition, it is expressed in the developing kidney, skeletal apparatus, and bone marrow, but is not essential for physiological homeostasis [75,78,79]. The HS chains of GPC1 can contribute to its functions by interacting with a wide range of molecules such as enzymes, cytokines, and growth factors [75]. In its function as a co-receptor, the GPC1 protein interacts with various signaling pathways such as FGF, Hh, TGF-β, and Wnt [78]. Heparin-binding mitogenic growth factors utilized GPC1 as a co-receptor; in addition, GPC1 exerted multiple roles in adhesion and cell growth [75,80]. During neuronal development, GPC1 plays a key role in axon guidance [81]. GPC1 is involved in the tumorigenesis of several tumor types, including PDAC, and several reports suggest a link between GPC1 and neurodegenerative diseases such as Alzheimer’s disease, prion disease, and Niemann–Pick-type C1 disease [18,38,59,60,82,83,84,85].

### 2.3. Glypican-1 in PDAC

As mentioned above, GPC1 is highly expressed in adult tissues and under physiological conditions exclusively in the testis, whereas its expression is low or absent in other tissues [18]. The high expression of GPC1 associated with PDAC has been widely reported in the literature [38,39,40,41,86].

GPC1 is highly expressed in PDAC tissues at both the mRNA and protein levels, whereas it is absent or very low in normal pancreas and chronic pancreatitis [38,39]. Northern blot analysis showed that mRNA expression of GPC1 was 8-fold higher in cancer tissues than in chronic pancreatitis and normal pancreas tissues. Western blot analysis showed that the GPC1 protein was present in four of six PDAC samples, whereas it was undetectable in normal samples. Immunohistochemistry showed a weak GPC1 signal in cells within the tumor, but a strong GPC1 signal was observed in fibroblasts surrounding the cancer cells. It is likely that the GPC1 signal observed in the fibroblasts could originate from the cancer cells. In situ hybridization revealed that GPC1 mRNA expression levels were high in cancer cells and adjacent fibroblasts [38].

Immunohistochemical studies showed that GPC1 was not present in the normal pancreas, that GPC1 was undetectable in most chronic pancreatitis samples, and that GPC1 staining was very weak in adjacent normal tissues. GPC1 was detected in 111 (59.7%) of 186 PDAC specimens examined, including liver, abdominal, and lymph node metastases [39]. Thus, GPC1 is highly expressed in PDAC and metastatic tissues compared with normal tissues and chronic pancreatitis [39]. GPC1 expression in PDAC appears to be associated with promoter hypomethylation, as GPC1 mRNA levels in PDAC samples are inversely proportional to DNA methylation [39]. Interestingly, GPC1 expression appears to be sex-specific, as most GPC1-positive cases are male [39]. High GPC1 levels have been associated with poor pathological differentiation and larger tumor masses [39]. In addition, GPC1 may be considered a prognostic factor for PDAC patients, as a correlation between high GPC1 levels and shorter overall survival has been observed in them [39].

The association between survival and the GPC1 expression level was further clarified by a study in which high GPC1 serum levels were associated with shorter overall survival compared to low GPC1 serum levels in PDAC patients [40].

In 2015, Melo et al. analyzed the sera of 190 PDAC patients. These sera had high levels of GPC1-positive circulating exosomes compared with healthy sera. The presence of these exosomes made it possible to distinguish with absolute specificity and sensitivity healthy individuals and individuals with benign lesions from PDAC patients. The level of GPC1-positive circulating exosomes was also associated with tumor burden and survival, with higher levels detected in distant metastases of patients. These results indicated that GPC1-positive circulating exosomes used as a diagnostic tool are better than carbohydrate antigen 19-9 (CA 19-9), the main tumor biomarker used in the clinic for PDAC patients [41].

GPC1-positive exosomes as a useful biomarker for PDAC patients were further investigated; specifically, 27 plasmas from PDAC patients and 16 plasmas from patients with benign pancreatic lesions were analyzed [86]. In this study, a high proportion of GPC1-positive exosomes was associated with a larger tumor size and tumor burden [86].

Several studies have demonstrated the active role of GPC1 in the progression of PDAC. In pancreatic cancer cell lines, GPC1 plays a key role in the mitogenic stimuli provided by fibroblast growth factor 2 (FGF2) and heparin-binding EGF-like growth factor (HB-EGF) [38]. Indeed, two pancreatic cancer cell lines (COLO-357 and PANC-1) were treated with phosphoinositide-specific phospolipase-C (PI-PLC), an enzyme responsible for the release of GPC1 from the cell membrane by GPI-anchor cleaving. This treatment abrogated the mitogenic effects of FGF2 and HB-EGF [38]. Subsequently, PANC-1 cells were transfected with an altered form of GPC1 that has a transmembrane domain in place of the GPI anchor and is thus immune to PI-PLC cleavage [38]. Replacement of the GPI anchor with a transmembrane domain restored the mitogenic effects that were lost due to the work of PI-PLC. In addition, GPC1 silencing reduced the mitogenic effects of FGF2 and HB-EGF [38].

Another contribution to the knowledge of the relationship between GPC1 and cancer progression, metastasis, and angiogenesis was made by Aikawa T. et al. [87]. Knockdown of GPC1 in the human pancreatic cancer cell line PANC-1 resulted in a reduction in tumor growth, metastasis, and angiogenesis compared to the PANC-1 cell line without knockdown [87]. After intrapancreatic injection of PANC-1 or T3M4 (human pancreatic cancer cell line), decreased angiogenesis and metastasis were observed in GPC1-null athymic mice compared with GPC1-positive athymic mice [87]. Moreover, these GPC1-null athymic mice showed little lung metastasis after intravenous administration of murine melanoma cells [87]. Another study confirmed the oncogenic role of GPC1 in PDAC, likely exerted through its interaction with the Hedgehog signaling pathway. Moreover, ecotropic viral integration site 1 (EVI1) appears to regulate GPC1 expression through miR-96 [88].

Alterations in TGF- β are thought to contribute to the pathogenesis of pancreatic cancer; indeed, the growth of COLO-357 was suppressed by TGF- β1 [89]. Of note, the silencing of GPC1 abrogated growth inhibition by TGF- β1, suggesting the key role of GPC1 in the TGF- β1 signaling pathway [89]. A few years later, the same authors performed work on Panc-1 and T3M4, two other pancreatic cancer lines, and showed that TGF- β, activin, and BMP signaling were inhibited by GPC1, albeit in a very minor manner [89]. Thus, it appears that GPC1 is not one of the major regulators of these signaling pathways [89].

In summary, the expression of GPC1 in adult tissues and under physiological conditions is restricted to the testis, while it is low or absent in other tissues [18]. Moreover, its localization on the cell surface, its overexpression, and its active role in the tumorigenesis of PDAC make GPC1 a promising therapeutic target for PDAC [18,38,59,60,61].

Taken together, the above-mentioned studies justify the feasibility of GPC1 as a specific target for PDAC.

## 3. Targeting Strategies

### 3.1. Antibody-Based Immunotherapy

In recent years, great efforts have been made in the field of tumor immunotherapy [16]. Immunotherapy has positively changed tumor medicine, and the benefits to patients since the introduction of this therapeutic approach into clinical treatment are obvious and promising [17]. An illustrative example is metastatic melanoma, where median survival has been extended from 8–12 months to 24 months thanks to this approach [17]. Ab-based-immunotherapy consists in the administration of monoclonal antibodies (mAbs) to treat tumors with a very specific strategy that reduces the side effects of conventional therapies [16]. In this direction, the identification of the tumor-associated antigen (TAA) is fundamental [16]. The antitumor activity resulting from mAbs administration is the result of different mechanisms depending on the following factors: antigen properties, target cell, and the interaction between the antigen-binding fragment (Fab) and the crystallizable fragment (Fc) of mAbs with the antigen and effector cells, respectively [90]. Therapeutic mAbs exert their anti-tumor activity mainly through two different mechanisms: (i) functional neutralization of the target antigen and (ii) opsonization of tumor cells [90]. In the first case, depending on the cell typology in which the target antigen is present, mAbs can interfere with tumor cell proliferation and metastasis, act on stromal cells to remodel the tumor microenvironment, or regulate the immune response. In the second case, the opsonization of tumor cells is caused by immune effector cell recruitment or the complement system, or a combination of both [90].

### 3.2. GPC1 as Target for Antibody-Based Immunotherapy in PDAC

Because GPC1 is exposed on the cell surface and overexpressed in PDAC tissues, whereas it is not expressed or is expressed at low levels in other healthy tissues, it embodies the properties of TAA, which mAbs can potentially target [18,19]. From a functional point of view, GPC1 can be considered as a potential target for mAbs because it can interact with various growth factors, such as fibroblast growth factor 2 (FGF2), vascular endothelial growth factor (VEGF), heparin-binding EGF-like growth factor, and TGF-β [19]. These growth factors are known to contribute to tumor cell proliferation, metastasis, and angiogenesis [19]. Moreover, in the context of PDAC, GPC1 expression has been detected in cancer-associated fibroblasts (CAF), which secrete the stroma. The presence of the stroma makes the tumor microenvironment difficult to pass for a conventional therapeutic strategy and creates a state of immunosuppression that promotes tumor growth [19]. For these reasons, functional neutralization of GPC1 by specific mAbs could potentially provide benefits by interfering with tumor growth, metastasis, and angiogenesis, and remodeling the tumor microenvironment to be more responsive to treatment and reduce immunosuppression. Between 2016 and 2018, a first in-human study investigated the use of the anti-GPC1 Miltuximab radiolabbeled with Gallium-67 [91]. Miltuximab is an IgG1 chimeric anti-GPC1 that was derived from MIL-38, which in turn was subcloned by BLCA-38 [92]. The trial was conducted on 12 patients with advanced solid tumors (9 patients with prostatic cancer, 2 patients with pancreatic cancer, and 1 with bladder cancer) [91]. The study demonstrated the safety and tolerability of Miltuximab; in addition this trial leads the way for the next phase I study in which Miltuximab will be conjugated with Lutetium-177 for therapy and with Zirconium-89 for imaging [91].

### 3.3. CAR-T Cells

**Figure 4:**
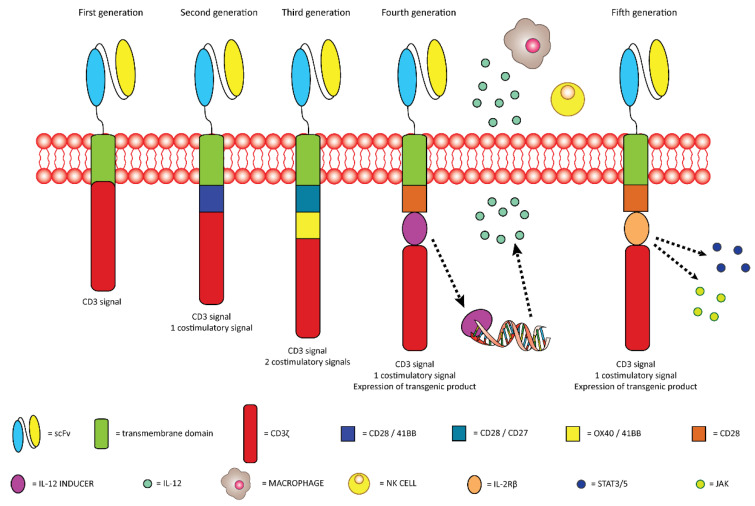
Schematic representation of the five generations of CAR-T cells.

The development of chimeric antigen receptor T (CAR-T) cells starts with the isolation of blood T cells from patients. These cells are subsequently tuned in order to express a receptor with the capability to recognize a specific TAA [93]. The general structure of CAR-T cells is composed of the molecule designated for the recognition of the TAA that, in most cases, is a single chain variable fragment (scFv), the spacer domain or hinge that provides both flexibility, enhancing the efficacy of the scFv, and a connection between the scFv and the transmembrane domain. The transmembrane domain is necessary for the communication between the scFv and the intracellular domain that is the one necessary for the organization of the response against the target cells [94]. To date, five generations of CAR-T cells, based on the organization of the intracellular domain, have been developed (Figure 4) [95].

The first generation is made of CD3ζ as a single intracellular domain [95]. In comparison with the first generation, the second generation presents the addition of the CD28 or 4-1BB as a costimulatory domain [93]. In the third generation two costimulatory domains are joined with the CD3ζ at the intracellular domain; the costimulatory domains can be represented by CD28, 4-1BB, CD27 and OX40 [95]. The fourth generation, also called TRUCKs (T cell Redirected for Universal Cytokine Killing), is characterized by the presence of CD28 as a costimulatory domain, the CD3ζ, but it also has the possibility to release chemokines such as IL-12 that can improve the activity of the CAR-T cells against antigen-negative cells by the recruitment of macrophages and NK cells [93]. Like the fourth generation, the fifth generation contains three intracellular domains composed of the CD3ζ, a costimulatory domain like CD28, and it has also the possibility to cooperate with the transcription factor STAT3 by its interaction with a truncated IL-2 receptor β-chain domain [96]. The cooperation, after the antigen binding, of these three domains triggers the cytokine (JAK-STAT3/5) signaling, leading to the full activation and proliferation of T cells [96]. Even if the use of CAR-T in the field of hematological tumors showed promising improvement in the clinical management of the patients, in the context of solid tumors, some problems have been observed like the on-target off-tumor fatal toxicities, loss of expression of target molecules by cancer cells, and deficient activation of perfused CAR-T cells in tumor sites [97]. In this context the use of GPC1, taking advantage of its peculiar characteristics of cellular localization and confined expression mainly in the PDAC tissue, could result in a promising improvement in the CAR-T efficacy and toxicological profile on healthy tissues. In 2020 Kato and colleagues developed, starting from the variable region of a specific anti-GPC1 mAb, GPC1-specific CAR-T cells that recognize mouse GPC1 and GPC1-specific CAR-T cells that recognize mouse GPC1 [97]. These two CAR-T cells were tested in terms of antitumor efficacy and toxicological effect in three different mouse models: immunocompetent syngeneic murine model of mouse sarcoma, immunocompetent syngenic murine model of mouse colon adenocarcinoma, and immunodeficient xenograft murine model of human esophageal squamous cancer [97]. The models showed antitumor effects without important side effects, but the syngeneic model provided some additional and more interesting information, given the possibility to evaluate the interaction with the mouse immune system. In detail, this model demonstrated an improvement in the activation of cytotoxic lymphocytes and the establishment of an immunological memory versus TAA different from GPC1 [97].

### 3.4. Antibody-Drug Conjugate

An antibody-drug conjugate (ADC) is a novel class of pharmacological compounds that take advantage of the specificity of an mAb to focus the toxicity of the anti-cancer drug at the tumor site [98]. The pioneer of ADC was the scientist Paul Ehrlich, who almost 100 years ago suggested the possibility of creating an antibody–toxin complex to improve toxin specificity [99]. In 1983, the first article regarding the use of ADC for patients with advanced ovarian and colorectal cancer was published [100]. The recent approvals in the clinical management of several cancers prompted the discovery of a novel structure of ADC [101,102,103,104,105,106,107]. It has been highlighted that the linkers play an essential role in the control of the off-target toxicity [99]. Linkers must have the capability to maintain the cytotoxic molecule attached to the mAb until the achievement of the final target [99]. Linkers can be classified as noncleavable and cleavable. Noncleavable linkers provide the release of the cytotoxic payload after the degradation of the mAb by lysosomal enzymes. Cleavable linkers are the most employed; the cleavage of the linker can occur by a pH change, a redox potential, or by a specific lysosomal enzyme [99]. The cytotoxic payload may have a cytotoxicity action even at low concentrations and a high stability in lysosomes and in blood circulation. In addition, long half-life, low molecular weight, low immunogenicity, and the chemical structure are aspects to be considered during the design of a novel ADC [99]. The two major families of cytotoxic payloads are the microtubule-disrupting agents (auristatin and maytansinoids) and the DNA-damaging agents (calicheamicin, duocarmycin, and doxorubicin) [99]. Another fundamental decision is the choice of the TAA to be targeted, and hence the mAb to use. In this direction, as mentioned in the above paragraph, the presence of GPC1, taking advantage of its peculiar characteristics of cellular localization and confined expression mainly in the PDAC tissue, could be exploited by the employment of a specific anti-GPC1 mAb representing a valid alternative for several solid tumors including PDAC. In 2020, Nishigaki and colleagues developed a novel ADC composed of an anti-GPC1 mAb and the monomethyl auristatin F (MMAF) [108]. The ADC was tested on in-vitro and on in-vivo murine models of PDAC, showing high anti-cancer activity in cell lines with high levels of GPC1 compared with those expressing GPC1 at low levels [108]. The ADC demonstrated anti-tumor activity in both the xenograft models and in the PDX, even if the anti-tumor activity is higher in the xenograft murine model, the levels of GPC1 being more homogeneous in comparison with the PDX model [108]. In 2021, Munekage and colleagues designated a novel ADC based on a humanized anti-GPC1 mAb bound to the monomethyl auristatin E (MMAE) [98]. This study reinforces the data from Nishigaki, showing a higher in-vitro antitumor activity of the ADC in GPC1-positive cell lines compared to those GPC1-negative [98]. The ADC also demonstrated high anti-tumor activity in the in-vivo xenograft models, in PDX murine models of PDAC, and in esophageal squamous cell carcinoma (ESCC) [98].

### 3.5. Nanoparticle-Based Target Therapy

**Figure 5:**
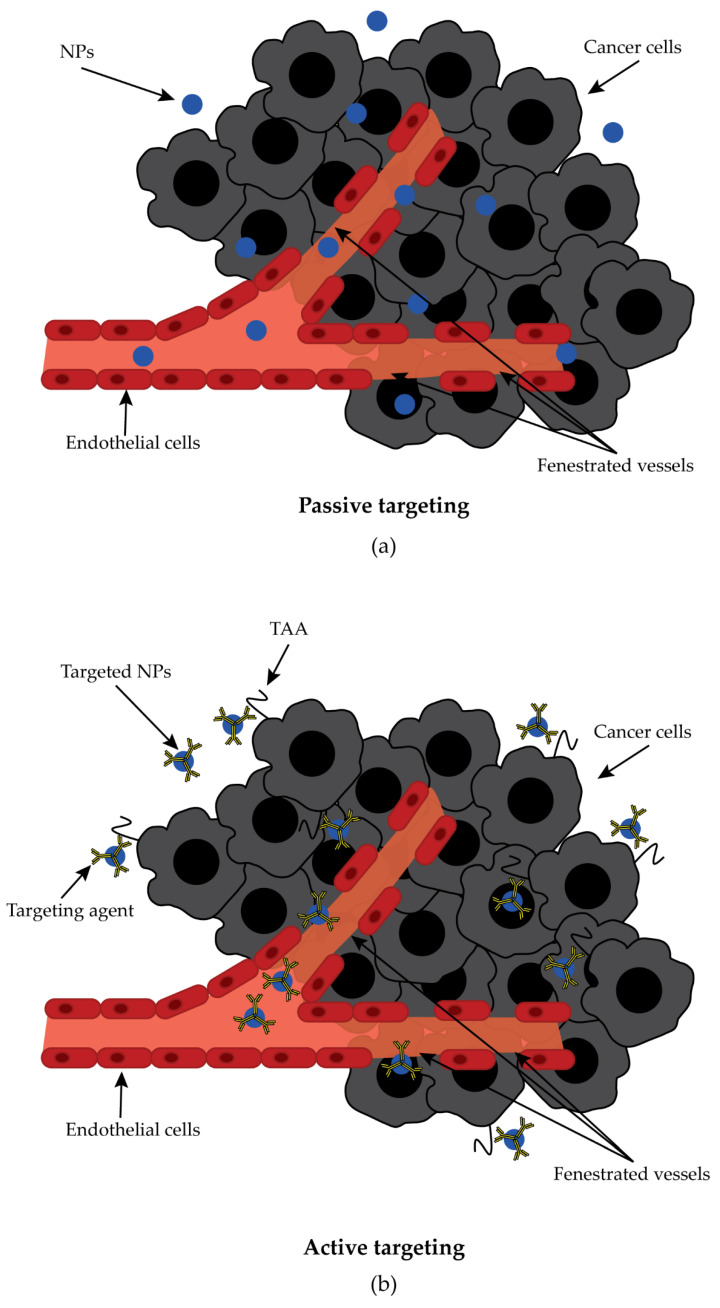
Panel (**a**): Passive targeting. NPs extravasate from blood circulation through the fenestrated vessels present in the leaky vasculature of a tumor, taking advantage of the EPR effect. Panel (**b**): Active targeting. NPs reach the tumor site through EPR and interact with the TAA through the targeting agent bound on the NPs surface.

The use of nanotechnology devices, especially nanoparticles (NP), has shown tremendous potential for cancer treatment [109,110,111]. These nanoformulations showed improved biodistribution, efficiency, selectivity, and reduced side effects compared to conventional free drugs [109,112]. In addition, they act as a protective shell that prevents rapid degradation and excretion of the drug and allows enhanced delivery of the drug to the desired treatment site [109,112]. Tumors have a particular microenvironment, a leaky and irregular vasculature with a lack of basement membranes and fenestrations ranging 200 nm to 2000 nm in combination with an impaired lymphatic system, which enables the so-called enhanced permeability and retention (EPR) effect. The EPR effect allows NPs to extravasate into tumor tissue, slowing their clearance through the lymphatic system and thus increasing their accumulation [110,113]. Drug delivery therapies can take advantage of this situation, especially when larger NPs are used. Indeed, small NPs can extravasate through both fenestrated and normal vessels and reach healthy organs, whereas larger NPs leave blood vessels mainly through fenestrations in tumor tissue and accumulate at the target site [109,114]. However, off-target accumulation could occur in organs that have a naturally fenestrated vasculature, such as the liver and spleen. To further increase the accumulation of NPs in tumor tissues, their surface can be modified and coated with targeted agents that redirect the nanoformulations to specific cell surface receptors or TAA (Figure 5) [109,110,111,114].

In the case of PDAC, its stroma acts as a barrier to therapeutic agents, an impairment that can be overcome by the use of NPs, increasing the permeability, retention and accumulation of anticancer drugs at the tumor site [115]. Several studies have investigated the use of NPs to target PDAC cells or, in some cases, to remodel the tumor microenvironment [116,117,118,119,120]. In in vitro experiments, they all showed a biocompatible profile when empty and an enhanced effect against PDAC cell lines when loaded with chemotherapeutic agents, mostly gemcitabine, or siRNA, compared to their free counterparts [116,117,118,119,120,121,122]. The synergistic effect of NPs loaded with chemotherapeutic agents and siRNAs was also investigated, showing a greater effect compared to single-agent treatment [117,118,119]. In vivo studies confirmed the positive in vitro results, with the prolongation of drug circulation time, inhibition of tumor growth with, in some cases, necrotic areas, and impaired tumor progression [117,118,119,120,121,122]. Interestingly, Confeld and colleagues used an iRGD peptide as a targeting agent to redirect gemcitabine and napabucasin (STAT3 inhibitor)-loaded NPs to PDAC cells and, in particular, to cancer stem cells overexpressing the neuropilin-1 receptor [121]. The addition of the targeting agent increased the uptake of the NPs by the cells and consequently the cellular cytotoxicity. In the orthotopic BXPC3 mouse model, it was demonstrated that therapy with NPs containing iRGD as a targeting agent suppresses tumor growth compared to treatments with a free drug or saline. In addition, the combination of gemcitabine and napabucasin in the iRGD NPs showed reduced tumor size at the end of the study, suggesting a synergistic effect of the two drugs [121].

### 3.6. Anti-GPC1 NPs

As previously described, GPC1 is a characteristic protein of PDAC, and therefore, its expression on the surface of tumor cells can be exploited by using NPs coated with an anti-GPC1 mAb [38]. Inorganic NPs targeting the GPC1 protein have been investigated for both imaging and therapy [123,124]. Interestingly, Qiu and coworkers showed an increased cellular uptake of Au NPs in PDAC cell lines with anti-GPC1 antibodies on the surface compared to those lacking the targeting agent [123]. In addition, magnetic resonance imaging in nude mice with an orthotopic PDAC tumor derived from BXPC3 showed a greater signal to the tumor site when the mice were treated with targeted Au-NPs compared with mice injected with Au-NPs without the anti-GPC1 antibody. They also tested in vivo the antitumor effect of Au-NPs loaded with oridonin, a compound with antitumor activity, and carrying or not carrying the anti-GPC1 antibody on their surface. Mice treated with the targeted NPs showed greater inhibition of tumor growth than those treated with Au-NPs without the anti-GPC1 antibody. Of note, no damage was observed in other organs [123].

### 3.7. Nanobubbles in PDAC

**Figure 6:**
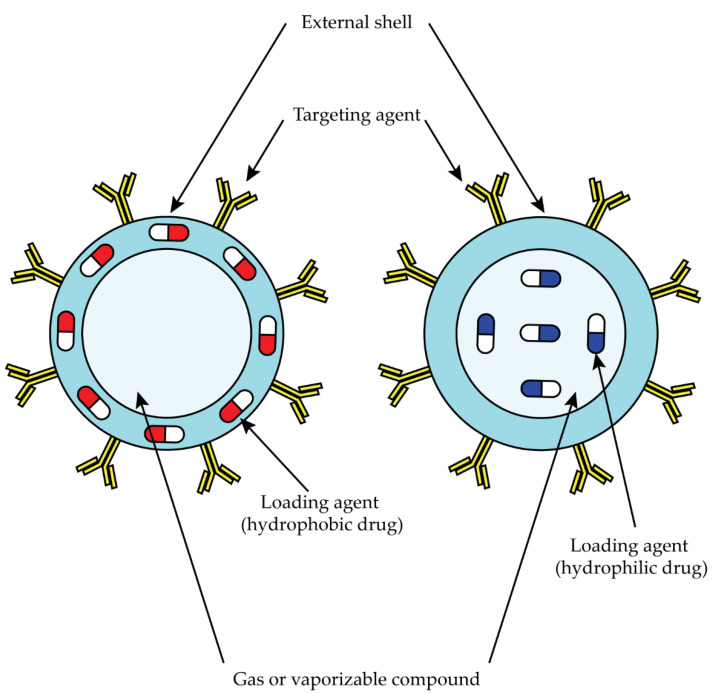
Schematic representation of NBs for drug delivery. Hydrophilic drugs are incapsulated in the external shell, and hydrophobic drugs are incapsulated in the gas core.

Nanobubbles (NBs) are a subgroup of NPs and consist of an outer biodegradable shell of phospholipids, polymers, or proteins and an inner core of a vaporizable compound or gas, usually perfluorocarbons or sulfur hexafluoride (Figure 6) [125,126,127].

Thanks to the phenomenon of acoustic cavitation, they are commonly employed for tumor imaging acting as ultrasound (US) contrast agents. However, NBs can be used not only for imaging but also for drug delivery. In fact, drugs encapsulated inside the NBs are protected from intracellular reactions, and the NBs themselves show stability, a prolonged circulation time, and can reach the tumor site through both the EPR effect and active targeting. Moreover, the use of external stimuli such as US or extracorporeal shock waves (ESW), can increase the rate of drug release in tumor tissue, thus enhancing the antitumor effect [125,126,127,128,129]. The use of NBs for imaging and/or treatment of PDAC was tested in some studies with interesting results [130,131,132]. Yang and colleagues used lipid NBs coated with IR-780 iodide, an agent used for photothermal and photodynamic therapy which showed spontaneous accumulation at tumor sites, to treat in-vitro PDAC cells and PDAC tumors in a mouse model. They demonstrated that IR-780-NBs were able to target PDAC cells both in vitro and in vivo; in addition, the use of docetaxel as a loading agent increased the therapeutic effect of the NBs acting synergistically as a photothermal and drug delivery system [130]. NBs were also used to deliver oxygen through the oral route in order to reduce tumor hypoxia, a condition that was correlated with cancer progression. Oxygen-NBs were administered through gavage in mice presenting a subcutaneous BXPC3 tumor. The treatment reduced the expression of the hypoxia-inducible-factor-1α (HIF-1α) and of the VEGF at the tumor site at both the transcriptional and translational level, suggesting a possible improvement for radiotherapy treatment [131]. Another study used NBs conjugated with liposomes loaded with paclitaxel to treat different cancer cells, including the PDAC cell lines MiaPaCa-2 and Panc-1. The authors exploited the ability of NBs subjected to US stimulus to cause sonopore formation at the cell membrane, increasing its permeability, in order to enhance the internalization of liposomes carrying the paclitaxel drug. The formulation of NBs plus liposomes loaded with paclitaxel and subjected to external US showed the greatest cellular cytotoxicity, with a 1000-fold increase compared to free paclitaxel, a 10-fold for MiaPaCa-2, and 100-fold for Panc-1, but decreased in the IC50 compared to liposome loaded with paclitaxel [132].

### 3.8. Chitosan and Chitosan Nanobubbles

In the above section, the outer shells of NBs for the treatment of PDAC were mainly composed of lipids [130,131,132]. Another possibility is the use of polymers, of which chitosan is widely used [127]. Chitosan is obtained from the deacetylation of chitin, an abundant natural polymer found in the cell walls of fungi, in the shells of crustaceans, and in some other structures of fish and invertebrates [133,134,135]. It is a linear polysaccharide with a positive charge and several deacetylated and acetylated units rich in hydroxyl (−OH) and amine (−NH2) groups, which are used for the addition of crosslinking agents. Chitosan is non-toxic, biocompatible, and biodegradable, and has been approved by the Food and Drug Administration (FDA) for drug delivery and tissue engineering [133,134,135]. Since chitosan is positively charged, it can interact with the negative charge of cell membranes through electrostatic interactions, resulting in increased cellular uptake, an important feature for drug delivery systems [136]. Thanks to its promising properties, chitosan has already been used as a matrix polymer for NBs (CS NBs) to achieve two different goals: NBs for the delivery of nucleic acids, such as DNA, antisense oligonucleotides (AON), or siRNA, or NBs for cancer treatment [128,129,137,138,139,140,141]. In the first case, CS NBs were used as transfection agents in conjunction with US or external shock waves (ESW), which resulted in the enhanced transfer of DNA and siRNA into cells, whereas they showed impairment in AON delivery [138,139,140]. In the second case, folate-coated CS NBs were used as smart bombs targeting folate-receptor-positive cells [141]. Folate-CS NBs reached the inside of the cell through endocytosis, and once there, the addition of US triggered their explosion, resulting in a drastic decrease in cell viability. This effect was confirmed in in vivo models, where the combined treatment destroyed the target tumor cells and significantly prolonged the overall survival of mice-bearing tumors without affecting other organs. Another way to use CS NBs for cancer treatment was to load the NBs with doxorubicin to treat anaplastic thyroid cancer cells both in vitro and in vivo and breast cancer cells in vitro [128,129,137,141]. Marano and colleagues demonstrated the interesting potential of CS NBs loaded with doxorubicin and triggered by ESW to exert a cytotoxic effect on anaplastic thyroid cancer cell lines. ESW were able to induce the intracellular release of doxorubicin, resulting in high drug levels in the nucleus, an effect that was also possible without the external stimulus, but to a lesser extent [129]. They also studied the formulation in an in vivo mouse model, which showed greater accumulation of doxorubicin at the tumor site compared to the same treatment without ESW. The combination therapy resulted in a reduction in tumor volume and weight without the cardiac toxicity typical of free doxorubicin [128]. Zhou and coworkers instead studied CS NBs for the treatment of breast cancer using an in vitro approach on the MCF-7 cell line. The addition of US resulted in the release of doxorubicin from inside the NBs into the MCF-7 cells and affected their viability to a greater extent than free doxorubicin combined with US [137].

## 4. Conclusions

The incidence of PDAC is increasing [6,7]. Currently available cytotoxic therapies for advanced disease are only moderately effective [14]. In oncology, monoclonal antibody-based immunotherapy enables the very specific treatment of tumors by reducing the toxic effects of conventional chemotherapy [16]. The large number and variety of potential antibody-based “targeted” approaches reflects the unique versatility of antibody-based platforms for cancer therapy development. In this context, the identification of specific tumor-associated antigens is essential [16]. In recent years, many agents have been used to realize this fundamental idea, and to date, several mAbs represent a valid therapeutic option for more cancer patients [17]. GPC1 appears to be a useful TAA for antibody-based therapies against PDAC because it is specifically overexpressed in PDAC tissues [18,19]. Considering the functions that the GPC1 protein performs in the context of the tumor cell and tumor microenvironment, the functional neutralization of GPC1 by a specific mAb would provide benefits for reducing tumor growth, metastasis, and angiogenesis, as well as remodeling the tumor microenvironment, making it more susceptible to treatments and reducing the state of immunosuppression [18,19]. Therefore, immunotherapies with effective anti-GPC1 mAbs could improve the clinical management of patients with PDAC. The first clinical evidence about the use of an anti-GPC1 mAb was published in 2021 [91]. During this trial, the anti-GPC1 Miltuximab was radiolabbeled with Gallium-67 and showed a safe toxicological profile supporting the establishment of the next phase I study for the use of Miltuximab as theranostic agent [91]. The promising results observed in the treatment of hematological malignancies using CAR-T cells prompt towards the introduction of this therapeutic strategy in the field of solid tumor as well. Unfortunately, the use of CAR-T to treat solid tumors showed some limitations derived by the onset of on-target off-tumor lethal toxicities, loss of antigen expression, and deficient activation of CAR-T cells. As reported in a promising pre-clinical study published in 2020 by Kato and colleagues, taking advantage of the peculiar characteristic of GPC1, as PDAC TAA, could represent a valid alternative to overcome the limitations onset during the earliest efforts regarding the use of CAR-T cells in of solid tumors. Another possibility to take advantage of the characteristics of GPC1 using an mAb is to conjugate it with an anti-cancer drug to obtain an ADC. In this case, the specificity of an mAb is coupled with the toxicities of an anti-cancer drug to focus the activity of the drug at the tumor site. In this direction, two interesting preclinical studies were performed in PDAC and ESCC in-vitro and in-vivo models using an anti-GPC1 conjugated with monomethyl auristatin F and monomethyl auristatin E, respectively [98,108]. Both of the ADCs showed promising activity in-vitro and in the in-vivo models [98,108]. These results provide encouraging evidence for the set-up of an ADC recognizing GPC1 to be transferred to the clinical management of PDAC patients and others with GPC1-expressing tumors. The encapsulation of antitumor drugs in nanotechnology devices, such as polymeric nanoparticles, allows for limiting toxicity to healthy tissues and concentrating the drug at the tumor site, preserving it without exposure to possible degradation in the bloodstream and increasing its solubility [109,112]. Conventional antitumor treatments such as radiotherapy and chemotherapy pose several clinical problems due to the high toxicity in healthy tissues and the development of resistance due to tumor heterogeneity [12]. In the clinic, some compounds based on nanotechnologies have been proposed to improve the pharmacokinetics and distribution of chemotherapeutic agents, such as nab-paclitaxel and doxyl [142]. However, in both cases, antibody conjugation has not yet been used. The development of nanotechnology approaches loaded with anticancer drugs (e.g., doxorubicin, paclitaxel) that can penetrate exclusively into the tumor thanks to active targeting mechanisms, such as conjugation with mAbs that recognize the GPC1, expressed mainly on the surface of malignant cells, would allow for focusing the toxicity exclusively to the tumor limiting the toxicity on healthy tissues and the development of resistance. Further information is needed to identify the best option for the structure of antibodies and polymeric nanoparticles that will allow for efficient drug release in the PDAC tumor.

## Figures and Tables

**Table 1:** PDAC prognosis and treatment by stage [14].

Disease Extent	Localized		Advanced	
Major vasculature involvement	Uninvolved or abutted	Uninvolved or abutted	Encased	Distant metastasis, irrespective of the major vascular involvement
Clinical stage	Resectable	Borderline resectable	Locally advanced	Metastatic
Prevalence of pancreatic cancer among patients newly diagnosed with PDAC, %	10–15	30–35	30–35	50–55
American Joint Committee on Cancer tumor, node, and metastasis stage	I–II	II–III	II–III	IV
Treatment intent	Curative	Curative	Supportive and palliative	Supportive and palliative
Treatment	Surgery plus adjuvant systemic therapy	Neoadjuvant systemic therapy; Surgery for resectable patients from favorable response; radiation for unresectable patients without distant metastasis	Neoadjuvant systemic therapy; Surgery for resectable patients from favorable response; radiation for unresectable patients without distant metastasis	Systemic therapy
5-years survival rate, %	35–45	10–15	10–15	<5

## Data Availability

Not applicable.
